# The Association Between Social Support and Psychosomatic Status in Cancer Patients During Mid-Course Radiotherapy: The Chain Mediating Roles of Psychological Capital and Coping Styles

**DOI:** 10.3390/healthcare14142138

**Published:** 2026-07-16

**Authors:** Huarui Yin, Liugang Gao, Xinye Ni

**Affiliations:** Department of Radiotherapy, The Affiliated Changzhou No. 2 People’s Hospital of Nanjing Medical University, Changzhou 213003, China; yinhuarui1@126.com (H.Y.); gaoliugang@126.com (L.G.)

**Keywords:** cancer radiotherapy, mid-course radiotherapy, social support, psychological capital, coping styles, psychosomatic status

## Abstract

**Background:** Cancer patients undergoing mid-course radiotherapy frequently experience psychosomatic distress, yet the psychosocial correlates and indirect pathways associated with this phenomenon remain insufficiently understood. **Objective:** To examine the direct and indirect associations between social support and psychosomatic status through psychological capital and coping styles in cancer patients undergoing mid-course radiotherapy. **Methods:** A cross-sectional study was conducted among 327 cancer patients recruited from the radiotherapy center of a tertiary-level general hospital between August 2025 and February 2026. Questionnaires were administered during the mid-course of radiotherapy, defined as the point at which the cumulative radiation dose reached 50% of the planned total dose. The Multidimensional Scale of Perceived Social Support (MSPSS), the Positive Psychological Capital Questionnaire (PPQ), the Cancer Coping Modes Questionnaire (CCMQ), and the Psychosomatic Status Scale for Cancer Patients (PSSCP) were utilized for assessment. Higher MSPSS, PPQ, and CCMQ scores indicate higher perceived social support, higher psychological capital, and more frequent use of the corresponding coping style, respectively, whereas higher PSSCP scores indicate poorer psychosomatic status. **Results:** (1) Social support, psychological capital, and positive coping scores were significantly negatively correlated with PSSCP scores, whereas negative coping scores were significantly positively correlated with PSSCP scores. (2) Structural equation modeling showed that social support was directly associated with psychosomatic status and indirectly associated with psychosomatic status through the independent and chain mediating roles of psychological capital and coping styles. **Conclusions:** Among cancer patients undergoing mid-course radiotherapy, higher levels of social support were associated with better psychosomatic status. This association was partially mediated by psychological capital and coping styles, both independently and sequentially. Given the cross-sectional design, these mediation findings should be interpreted as association-based. The findings highlight the potential importance of integrating social support enhancement with interventions targeting psychological resources and coping behaviors to support psychosomatic well-being during radiotherapy.

## 1. Introduction

Cancer remains one of the leading causes of morbidity and mortality worldwide. In 2022, there were approximately 20 million new cancer cases and 9.7 million cancer-related deaths globally, and both incidence and mortality rates continue to rise [1]. Radiotherapy is a critical component of cancer treatment, particularly for local tumor control [2]. However, although effective, radiotherapy frequently induces adverse effects. Patients may experience somatic symptoms such as pain, nausea, vomiting, and fatigue [3], alongside significant psychological distress including anxiety and depression [4]. These problems may be associated with poorer psychosomatic status, reduced treatment adherence, delayed recovery, and diminished therapeutic efficacy. In this study, psychosomatic status refers to patients’ integrated psychological, somatic, social-functioning, and psychological-behavioral condition during cancer treatment, which is conceptually broader than distress or symptom burden but more specific than overall quality of life. The stressors experienced by patients undergoing radiotherapy extend beyond disease-related factors and include treatment-setting-specific environmental demands, such as confined treatment spaces, equipment noise, immobilization, and passive positioning, as well as the cumulative burden associated with repeated treatment sessions. These setting-specific demands may be associated with psychological distress, physical discomfort, and disruption of daily social functioning [5,6,7,8]. Therefore, this study adopts an integrative psychosomatic perspective to investigate the psychosomatic state of cancer patients during mid-course radiotherapy and potential psychosocial pathways, with the aim of providing insights into psycho-oncology and integrative healthcare. Accordingly, the PSSCP was selected because it is a cancer-specific instrument developed and validated in Chinese cancer populations and assesses psychosomatic status across psychological, somatic, social-functioning, and psychological-behavioral dimensions, which is consistent with the multidimensional focus of the present study.

Among the myriad factors influencing the psychosomatic state of radiotherapy patients, social support serves as a significant protective factor for their psychosomatic well-being [9]. According to the main effect model, social support is universally beneficial for mental health and reduces negative affect [10]. Both domestic and international research has confirmed that social support improves psychosocial adjustment in patients following head and neck radiotherapy [11], reduces symptoms of depression and anxiety in cervical cancer patients [12], and alleviates distress and anxiety during radiotherapy through psychological bolstering [13]. However, other findings have indicated that breast cancer patients undergoing radiotherapy who possess high intelligence but low social support exhibit higher levels of depression [14]. Although these studies support the protective role of social support, two limitations remain. First, most have used single psychological indicators, such as anxiety or depression, as outcomes, without adopting an integrated psychosomatic assessment. Second, research specifically focusing on the mid-course of radiotherapy remains limited. Previous research and review evidence have examined psychosocial needs, distress, and quality of life before radiotherapy, during the overall treatment course, after radiotherapy, or during follow-up, whereas stage-specific psychosomatic assessment at the mid-course point, defined as approximately 50% of the planned radiation dose, remains insufficiently characterized [15,16]. Furthermore, it is essential to note that radiotherapy for head and neck tumors involves irradiation of the hippocampus and impairment of core functions such as speech and swallowing, mechanisms of psychosomatic impact that differ from those associated with tumors at other sites [17,18]. Consequently, the present study excluded head and neck tumors to reduce one important source of heterogeneity and focused on thoracic, abdominal, pelvic, breast, bone, and hematologic/lymphatic malignancies. As an external resource, social support may be associated with psychosomatic status both directly and indirectly through internal psychological resources and coping behaviors. Examining these pathways may inform the refinement of psychosocial interventions tailored for radiotherapy patients.

At the level of internal resources, psychological capital—defined as an individual’s positive psychological state of development characterized by self-efficacy, hope, resilience, and optimism [19]—constitutes a vital protective resource for maintaining mental health. Drawing upon Conservation of Resources (COR) theory [20], psychological capital, as a key psychological resource, assists individuals in managing stress and preserving psychological stability. For cancer patients in the mid-phase of radiotherapy, psychological capital may be reflected in a perceived sense of control over the treatment process (self-efficacy), positive expectations for recovery (hope and optimism), and the capacity to recover from side effects and uncertainty (resilience), and may be associated with better psychosomatic status. Research has explored the role of psychological capital in oncology populations. For instance, psychological capital combined with mindfulness promotes mental health among breast cancer patients undergoing chemotherapy [21]. Following the completion of radiotherapy, optimism (a core dimension of psychological capital) has been identified as a significant factor influencing better mental health outcomes [15]. Although these studies preliminarily support a favorable association between psychological capital and the mental health of cancer patients, most have focused on patients receiving chemotherapy or post-radiotherapy survivors. The role of psychological capital during the mid-course of radiotherapy, as well as its mediating role in the relationship between social support and psychosomatic status, has not been systematically examined. Moreover, social support may be associated with psychological capital by providing emotional comfort, information, and tangible assistance. Studies have shown that social support positively predicts psychological capital across diverse cancer populations [22,23]. Notably, among patients with multiple myeloma, social support and psychological capital have been found to synergistically enhance quality of life [24]. Accordingly, we proposed Hypothesis 1 (H1): psychological capital mediates the relationship between social support and psychosomatic status in patients undergoing mid-course radiotherapy.

Beyond internal resources, coping styles—the cognitive and behavioral efforts individuals employ to manage stress—also play a pivotal role in psychosomatic adaptation. Positive coping refers to adaptive responses such as problem-solving and positive reappraisal, whereas negative coping manifests as maladaptive reactions including avoidance, resignation, and fantasy [25]. According to COR theory [20], positive coping facilitates resource acquisition and conservation, whereas negative coping may exacerbate resource depletion and psychological burden. Current evidence indicates that coping styles are associated with mental health and quality-of-life outcomes in cancer patients; however, the direction and strength of these associations are not entirely consistent across studies. Macía et al. [26] reported that different coping strategies showed distinct relationships with mental health outcomes among people with cancer, suggesting that coping should not be regarded as uniformly protective. Similarly, Roszkowska and Białczyk [27] found that, among breast cancer patients undergoing radiotherapy, avoidant coping was negatively associated with mental, physical, and overall quality of life, whereas active coping showed only limited benefits. These inconsistent findings may be related to differences in cancer type, treatment phase, stress intensity, and the specific coping dimensions measured. Although previous studies have highlighted the importance of coping styles in oncology populations, two gaps remain. First, most studies have examined only the associations between coping styles and psychological outcomes, or have treated coping as a moderator, leaving its mediating role between external resources (e.g., social support) and psychosomatic outcomes underexplored. Second, the mediating pathway through which coping styles link social support to psychosomatic status has not been systematically tested. As a crucial external resource, social support may be indirectly associated with psychosomatic status through coping styles. The stress-buffering model of social support posits that social support is particularly relevant under high-stress conditions, with one proposed pathway involving the modulation of individual coping processes [10]. Research suggests that higher levels of social support are associated with greater utilization of positive coping and reduced reliance on negative coping [28]. Therefore, we proposed Hypothesis 2 (H2): coping styles (positive and negative coping) mediate the relationship between social support and psychosomatic status in patients undergoing mid-course radiotherapy.

Cancer patients undergoing radiotherapy confront multiple challenges, including physical suffering, psychological distress, and altered social roles [29]. Theoretically, the association between social support and psychosomatic status may involve a sequential process in which external support is linked to internal resources and behavioral responses. Research has demonstrated that individuals with higher levels of psychological capital are more inclined to adopt positive coping strategies and exhibit a negative association with negative coping [30]. Further studies have elucidated the pathways linking psychological capital and coping styles: among cancer patients, self-efficacy (a core dimension of psychological capital) and positive coping jointly mediate the association between social support and psychological resilience [31]. Among individuals with HIV/AIDS, positive coping partially mediates the association between psychological capital and depression [32]. These findings suggest that psychological capital may be associated with psychosomatic health outcomes through coping styles. Previous research has predominantly examined single mediating pathways and has seldom integrated “internal psychological resources” and “behavioral coping strategies” within a unified framework. Consequently, this study aimed to construct and test a serial mediation model to more systematically elucidate the psychosocial pathways associated with psychosomatic status in cancer patients undergoing mid-course radiotherapy. Accordingly, we proposed Hypothesis 3 (H3): psychological capital and coping styles (positive and negative coping) play serial mediating roles in the relationship between social support and psychosomatic status in patients undergoing mid-course radiotherapy.

## 2. Methods

### 2.1. Study Design and Participants

This cross-sectional study employed convenience sampling to recruit cancer patients undergoing radiotherapy at the Radiotherapy Center of a tertiary Grade A hospital between August 2025 and February 2026. The sampling frame consisted of cancer patients receiving radiotherapy at this center during the recruitment period who met the eligibility criteria and had reached the mid-course radiotherapy point. The study was conducted in accordance with the principles of the Declaration of Helsinki and was approved by the Ethics Committee of Changzhou No. 2 People’s Hospital of Nanjing Medical University (Approval No.: [2025]YLJSA041).

The inclusion criteria were as follows: (1) pathologically confirmed malignant tumor (stage I–IV); (2) stable medical condition, self-reported awareness of the cancer diagnosis, and receipt of radiotherapy at the study hospital (awareness was assessed before questionnaire administration by asking patients whether they had been informed of their cancer diagnosis by their physician or family members); (3) age ≥ 18 years; (4) intact cognitive function and ability to cooperate with survey completion; (5) provision of informed consent to participate in the study. The exclusion criteria were as follows: (1) presence of severe comorbidities (e.g., heart failure, stroke); (2) cognitive impairment, history of severe mental illness, or inability to cooperate; (3) communication or literacy difficulties; (4) expected survival < 1 year; (5) evident disease progression or interruption of radiotherapy during the investigation period; (6) head and neck tumors. Patients with an expected survival of less than one year were excluded primarily for ethical and practical reasons. These patients may have greater clinical fragility, heavier symptom burden, and more urgent palliative or supportive care needs; therefore, participation in a questionnaire-based study during radiotherapy could increase unnecessary burden. This exclusion was not based on the assumption that psychosocial resources or social support are unimportant in this population. In accordance with the recommended subject-to-variable ratio for factor analysis and structural equation modeling, a minimum sample size of 200 cases was required [33].

### 2.2. Data Collection

All questionnaire surveys were administered during the mid-course of radiotherapy (defined as the point at which the cumulative radiation dose reached 50% of the total planned dose). Researchers provided eligible patients with a standardized explanation regarding the purpose and significance of the study. After obtaining informed consent, patients completed the closed-ended questionnaires on site after receiving standardized instructions from a trained researcher. Patients selected their own response options, and the researcher did not interpret or categorize their answers. Disease-related information was extracted from the patients’ medical records. To ensure data accuracy, two researchers independently entered the completed questionnaire data, and discrepancies were resolved by checking the original questionnaires. The final 327 valid questionnaires had no item-level missing data; therefore, no statistical imputation was performed. A total of 345 questionnaires were distributed, and 327 valid questionnaires were returned, yielding an effective response rate of 94.78%.

### 2.3. Instruments

#### 2.3.1. Demographic and Clinical Characteristics Questionnaire

A self-designed questionnaire was used to collect information on patients’ gender, age, educational level, marital status, employment status, number of children, place of residence, monthly household income, type of medical insurance, cancer diagnosis, and TNM (Tumor–Node–Metastasis) stage.

#### 2.3.2. Multidimensional Scale of Perceived Social Support (MSPSS)

The Chinese version of the Multidimensional Scale of Perceived Social Support (MSPSS), originally developed by Zimet et al. [34] and translated and revised by Jiang, was employed [35]. The scale comprises 12 items encompassing three dimensions: family support, friend support, and support from significant others. Items are rated on a 7-point Likert scale (1 = strongly disagree, 7 = strongly agree). Higher total scores indicate higher levels of perceived social support. In the present study, the overall Cronbach’s α coefficient for the scale was 0.901.

#### 2.3.3. Positive Psychological Capital Questionnaire (PPQ)

The Positive Psychological Capital Questionnaire (PPQ), developed by Luthans and translated into Chinese by Zhang Kuo [36], was utilized. The questionnaire consists of 26 items covering four dimensions: self-efficacy, hope, resilience, and optimism. Responses are rated on a 7-point Likert scale (1 = strongly disagree, 7 = strongly agree). Items 8, 10, 12, 14, and 25 are reverse-scored. Higher scores reflect higher levels of psychological capital. In the present study, the Cronbach’s α coefficient for the questionnaire was 0.948.

#### 2.3.4. Cancer Coping Modes Questionnaire (CCMQ)

The Cancer Coping Modes Questionnaire (CCMQ), developed by Huang et al., was administered [25]. The questionnaire includes 26 items across five dimensions: confrontation, avoidance and suppression, resignation, fantasy, and venting. Items are rated on a 4-point Likert scale (4 = always, 3 = often, 2 = sometimes, 1 = never). Following the original dimensional structure of the CCMQ, the confrontation subscale was operationalized as positive coping, whereas avoidance and suppression, resignation, fantasy, and venting were operationalized as negative coping. In the present study, the positive coping score was represented by the confrontation subscale, and the negative coping score was calculated from the four negative coping subscales [25,37]. The Cronbach’s α coefficient for the positive coping dimension was 0.869, and that for the negative coping dimension was 0.935.

#### 2.3.5. Psychosomatic Status Scale for Cancer Patients (PSSCP)

The Psychosomatic Status Scale for Cancer Patients (PSSCP), developed by Chen, was used to assess patients’ psychosomatic status [38]. The scale comprises 16 items across four dimensions: psychological, somatic, social functioning, and psychological-behavioral plasticity. Items are rated on a 5-point scale, with items 5, 11, and 15 reverse-scored. Higher total scores indicate poorer psychosomatic status (i.e., greater psychosomatic disturbance), whereas lower scores indicate better psychosomatic status. In the present study, the Cronbach’s α coefficient for the scale was 0.916.

### 2.4. Statistical Analysis

Data analysis was performed using IBM SPSS Statistics, version 25.0, and IBM SPSS Amos, version 26.0 (IBM Corp., Armonk, NY, USA). Descriptive statistics were presented as frequencies and percentages for categorical variables and as means ± standard deviations for continuous variables after testing for normality. For normally distributed continuous variables, independent-samples t-tests and one-way analysis of variance (ANOVA) were used to compare PSSCP scores across demographic and clinical subgroups. Pearson correlation analysis was utilized to examine correlations among the primary variables. Multiple linear regression analysis was performed with the PSSCP total mean score as the dependent variable; demographic and clinical variables were entered as covariates, and social support, psychological capital, positive coping, and negative coping were entered as independent variables. Structural equation modeling was conducted using AMOS 26.0 to test the hypothesized indirect associations. In the SEM analyses, dimension-level mean scores were calculated according to the scoring rules of each instrument and entered into AMOS as observed indicators of their corresponding constructs. The structural paths were specified a priori to test the hypothesized associations among social support, psychological capital, coping styles, and psychosomatic status, including the chain indirect pathway from social support to psychosomatic status through psychological capital and coping styles. No modification-indices-driven adjustment of structural paths was performed. Model fit was evaluated using χ^2^/df, root mean square error of approximation (RMSEA), goodness-of-fit index (GFI), comparative fit index (CFI), incremental fit index (IFI), Tucker–Lewis index (TLI), and adjusted goodness-of-fit index (AGFI). Acceptable model fit criteria were defined as χ^2^/df ≤ 5, GFI/CFI/IFI/TLI/AGFI ≥ 0.90, and RMSEA < 0.08. The bias-corrected percentile bootstrap method with 5000 resamples was applied to generate 95% confidence intervals for indirect effects. An effect was considered statistically significant if the 95% confidence interval did not include zero. The significance level was set at α = 0.05 (two-tailed).

## 3. Results

### 3.1. Common Method Bias Test

Prior to data analysis, Harman’s single-factor test was conducted to assess potential common method bias (CMB) [39]. Sixteen factors demonstrated eigenvalues greater than 1, and the first factor accounted for 24.64% of the total variance, which is below the 40% threshold proposed by Hair et al. [40]. These results indicate that no serious common method bias is present in this study. These results suggest that no single dominant factor was evident; however, common method bias cannot be completely ruled out because all main variables were collected using self-report questionnaires at the same time point.

### 3.2. Multicollinearity Assessment

Multicollinearity was evaluated using the variance inflation factor (VIF). The VIF values ranged from 1.023 to 1.507, all of which fell below the widely accepted threshold of 5, indicating that no substantial multicollinearity exists among the variables.

### 3.3. Demographic and Clinical Characteristics of Participants

A total of 327 participants were enrolled, comprising 131 males (40.1%) and 196 females (59.9%). Univariate analysis revealed significant differences in PSSCP scores according to employment status (*t* = 2.01, *p* = 0.046), number of children (*F* = 2.98, *p* = 0.032), average monthly household income (*F* = 5.89, *p* = 0.003) and cancer stage (*F* = 2.76, *p* = 0.043). Specifically, patients who were employed, had more children, reported higher household income, or were diagnosed with earlier cancer stages exhibited lower PSSCP scores, indicative of relatively better psychosomatic status. No significant differences in PSSCP scores were observed for other demographic or clinical variables (all *p* > 0.05). Detailed information is presented in Table 1.

### 3.4. Descriptive Statistics and Correlation Analysis

Pearson correlation analysis (Table 2) revealed that scores for social support, psychological capital, and positive coping were all significantly negatively correlated with PSSCP scores (r ranging from r = −0.456 to −0.416, *p* < 0.01). Negative coping was significantly positively correlated with PSSCP scores (r = 0.360, *p* < 0.01) and significantly negatively correlated with social support and psychological capital scores. These findings indicate that patients who perceived more adequate social support, possessed higher levels of psychological capital, and employed more positive coping strategies exhibited better psychosomatic status (i.e., lower PSSCP scores). Conversely, patients who more frequently utilized negative coping strategies demonstrated poorer psychosomatic status (i.e., higher PSSCP scores).

### 3.5. Multiple Linear Regression

A multiple linear regression analysis was conducted with the PSSCP total score as the dependent variable. Variables that were statistically significant in the univariate analysis (employment status, number of children, average monthly household income, and cancer TNM stage) were entered as covariates, while social support, psychological capital, positive coping, and negative coping were entered as independent variables. The regression results showed that monthly household income (*β* = −0.092, *p* = 0.046), Social support (*β* = −0.183, *p* = 0.001), psychological capital (*β* = −1.153, *p* = 0.005) and positive coping (*β* = −2.237, *p* < 0.001) were significantly negatively associated with psychosomatic status. Negative coping (*β* = 0.142, *p* = 0.006) was significantly positively associated with psychosomatic status. The overall model explained 33.2% of the variance (adjusted *R*^2^ = 0.332, *F* = 21.24, *p* < 0.001). Detailed information is presented in Table 3.

### 3.6. Structural Equation Modeling and Mediation Effect Testing

**Figure 1:**
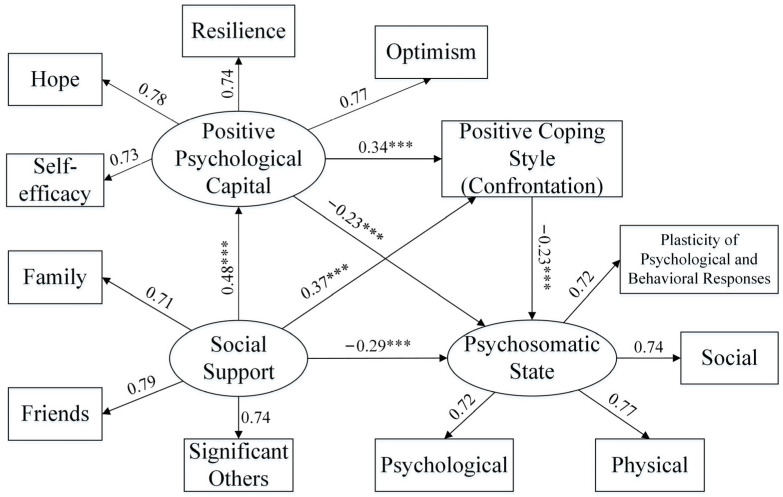
The association between social support and psychosomatic status in cancer patients: The mediating role of psychological capital and positive coping. Note: *** *p* < 0.001.

**Figure 2:**
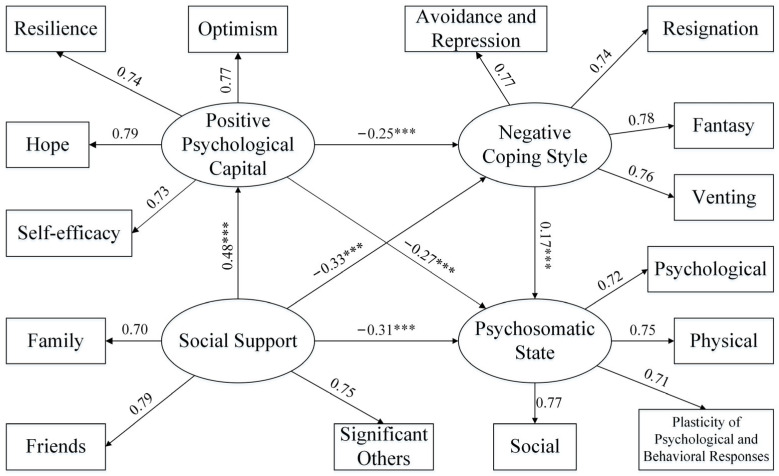
The association between social support and psychosomatic status in cancer patients: The mediating role of psychological capital and negative coping. Note: *** *p* < 0.001.

The measurement properties of the latent constructs were examined using standardized factor loadings, composite reliability (CR), and average variance extracted (AVE). In the positive coping model, standardized factor loadings ranged from 0.713 to 0.791, AVE values ranged from 0.543 to 0.574, and CR values ranged from 0.794 to 0.843. In the negative coping model, standardized factor loadings ranged from 0.701 to 0.793, AVE values ranged from 0.543 to 0.582, and CR values ranged from 0.794 to 0.848. These results supported acceptable reliability and convergent validity of the latent constructs. Structural equation modeling was employed to examine the pathways linking social support to psychosomatic status. For Model 1, the fit indices were as follows: χ^2^/df = 2.093, RMSEA = 0.058, GFI = 0.950, CFI = 0.966, IFI = 0.967, TLI = 0.954, AGFI = 0.920. For Model 2, the fit indices were as follows: χ^2^/df = 1.690, RMSEA = 0.046, GFI = 0.945, CFI = 0.972, IFI = 0.972, TLI = 0.964, AGFI = 0.922. The path diagrams are presented in Figure 1 and Figure 2. In Figure 1 and Figure 2, the coefficients from latent variables to their corresponding subscale indicators represent standardized factor loadings. These standardized factor loadings were all statistically significant and within an acceptable range, indicating that the observed subscale indicators adequately represented their corresponding latent constructs. The results of the bootstrap mediation effect testing (Table 4) indicated that the 95% confidence intervals for the total, direct, and indirect effects of social support on PSSCP scores did not include zero, thereby confirming significant mediation effects. In Model 1, the independent mediating effect of psychological capital was −0.112 (95% CI [−0.193, −0.048]), the independent mediating effect of positive coping was −0.085 (95% CI [−0.152, −0.037]), and the chain mediating effect of psychological capital and positive coping was −0.037 (95% CI [−0.068, −0.016]). The total indirect effect accounted for 44.9% of the total effect. In Model 2, the independent mediating effect of psychological capital was −0.128 (95% CI [−0.212, −0.068]), the independent mediating effect of negative coping was −0.057 (95% CI [−0.119, −0.011]), and the chain mediating effect of psychological capital and negative coping was −0.021 (95% CI [−0.051, −0.004]). The total indirect effect accounted for 39.53% of the total effect. Detailed information is provided in Table 4.

## 4. Discussion

### 4.1. Characteristics of Psychosomatic Status in Cancer Patients During Mid-Course Radiotherapy

In the present study, psychosomatic status among cancer patients assessed during mid-course radiotherapy differed according to employment status, number of children, monthly household income, and cancer stage. This finding indicates that psychosomatic status during this treatment phase was not uniform across patients and supports the need to identify subgroups who may be more vulnerable to poorer psychosomatic status. The assessment time point of this study, defined as the point at which the cumulative radiation dose reached 50% of the planned total dose, may represent a clinically meaningful checkpoint for psychosomatic assessment. This interpretation is consistent with previous evidence showing that cancer patients undergoing radiotherapy commonly experience psychosomatic distress [41]. Similarly, recent studies in radiotherapy populations have reported a high prevalence of psychosocial distress and dynamic changes in distress and quality of life during radiotherapy, further supporting the clinical relevance of psychosomatic assessment during active treatment [16,42]. Compared with previous studies, the level of psychosomatic distress among cancer patients at mid-course radiotherapy has been reported to be higher than that among patients with benign tumors but lower than that among lung cancer patients receiving chemotherapy [43]. This pattern may partly reflect differences in treatment mechanisms, as radiotherapy is primarily a local treatment, whereas chemotherapy is systemic and may involve more extensive toxic effects [44]. Longitudinal evidence also suggests that psychological distress varies across radiotherapy phases and tumor types: depressive symptoms in patients with head and neck cancer may gradually worsen during radiotherapy and peak shortly after treatment completion, whereas depression in gastrointestinal cancer may peak during mid-course radiotherapy, and psychological distress in esophageal cancer may initially increase and then decrease [45,46,47]. Together, these findings support the clinical relevance of focusing on mid-course radiotherapy as an important period for psychosomatic assessment and targeted supportive care.

Monthly household income is an important factor influencing patients’ psychosomatic status during radiotherapy, which is consistent with previous research [48]. Higher income may buffer against “financial toxicity” and alleviate economic pressure and psychological burden [49]. Employment status, number of children, and cancer stage were also associated with differences in psychosomatic status, suggesting that patients’ psychosomatic status is comprehensively influenced by multiple factors, including social roles, family structure, and disease severity.

Notably, psychosomatic status is not only relevant to patients’ subjective experience but may also substantially affect the precision of radiotherapy. One study showed that breast cancer patients undergoing radiotherapy who had pronounced somatic symptoms of anxiety were at higher risk of setup errors during radiotherapy [50]. This finding suggests that systematic assessment and support of psychosomatic status may be important not only for enhancing quality of life but also for helping maintain treatment quality and reducing the risk of radiotherapy-related errors. Therefore, clinical practice should place high importance on integrated psychosomatic assessment in radiotherapy patients and consider the role of socioeconomic factors in this process.

### 4.2. Potential Pathways Linking Social Support to Psychosomatic Status: The Chain Mediating Role of Psychological Capital and Coping Styles

#### 4.2.1. The Direct Favorable Association Between Social Support and Psychosomatic Status

In the present study, the level of social support among cancer patients at mid-radiotherapy was similar to that reported by lung cancer patients receiving chemotherapy [23]. Previous research has demonstrated the favorable association between social support and psychosomatic health on the psychosomatic health of chemotherapy patients. The present study extends this perspective to patients at mid-course radiotherapy and further explores the underlying mechanisms. Radiotherapy is typically administered once daily over several weeks, and its unique environmental stressors—confined spaces, immobilization, and mechanical noise—create a “highly mandatory, low-controllability” situation. In this context, when patients perceive stable engagement and support from healthcare providers and family members, social support serves as a “secure base” and a “helping system” for coping with stress [51]. Adequate social support satisfies belonging needs, buffers psychological distress, and enhances sense of control and treatment adherence—a relationship theoretically supported by the main effect model of social support [10].

#### 4.2.2. The Mediating Role of Psychological Capital: The Internalization Pathway of External Resources

The mediating role of psychological capital between social support and psychosomatic status is consistent with the core tenet of the conservation of resources theory—that external resources may be associated with the development of internal resources and better health-related outcomes [20,23,52]. Notably, the independent mediating effect of psychological capital was dominant in both models, suggesting that its role is robust across contexts, which is consistent with findings in breast cancer patients and other populations [53,54,55]. Psychological capital (self-efficacy, hope, resilience, and optimism), as an internal psychological resource, may be associated with patients’ positive expectations and sense of control when facing radiotherapy side effects, and may therefore be linked to lower psychosomatic distress. Patients at mid-radiotherapy often simultaneously experience distress from current side effects and uncertainty about subsequent treatment; this dual pressure suggests that cultivating psychological capital is particularly meaningful at this stage. Previous research has also shown that during the physically and psychologically stressful period of radiotherapy, social support is associated with psychosomatic status through individuals’ internal resources [56]. Psychological capital may therefore function as a key link through which perceived support is associated with self-efficacy, hope, optimism, and resilience, thereby showing a favorable association with psychosomatic status. Therefore, clinical interventions may prioritize the cultivation of psychological capital in patients undergoing mid-course radiotherapy.

#### 4.2.3. The Mediating Role of Coping Styles: The Translational Pathway of Behavioral Strategies

During mid-course radiotherapy, positive coping and negative coping play directionally opposite mediating roles between social support and psychosomatic status. As an important external resource, social support may be associated with more positive coping strategies and less negative coping, thereby being linked to better psychosomatic status. Existing research supports these pathways. Ren et al. [57] found that high levels of social support are associated with patient health outcomes through positive coping. Negative coping (e.g., avoidance, resignation), in contrast, is significantly positively associated with poorer psychological, physical, and quality-of-life outcomes [27,58]. A strong social support network may help patients reduce such maladaptive behavioral patterns [59]. During radiotherapy, psychological distress triggered by side effects such as local skin reactions and fatigue, as well as uncertainty about prognosis, often leads patients to social withdrawal [60,61]. In the absence of adequate social support, patients may be more likely to adopt maladaptive coping patterns such as avoidance and resignation, which may be linked to poorer psychosomatic status. Thus, coping styles represent an important mediating pathway linking social support and psychosomatic status, suggesting how external resources may be linked to psychosomatic well-being during the high-stress phase of mid-course radiotherapy through patients’ behavioral responses.

#### 4.2.4. The Chain Mediating Effect of Psychological Capital and Coping Styles: An Integrated Pathway from Resource Empowerment to Behavioral Transformation

This study provides evidence consistent with a chain indirect pathway involving psychological capital and coping styles in the relationship between social support and psychosomatic status among cancer patients at mid-course radiotherapy. A similar association pattern has been reported in other populations: among civil servants, social support is associated with coping styles through psychological capital [62]. Among cancer patients, social support is indirectly associated with psychological resilience through self-efficacy (a core dimension of psychological capital) and positive coping [31]. Building on previous research, the present study offers the following extensions. First, it focuses on a targeted patient population. Previous studies have mostly focused on patients undergoing chemotherapy, postoperative recovery, or those with a single type of cancer. In contrast, the present study specifically examines patients undergoing radiotherapy, who face unique stressors such as confined spaces, equipment noise, and immobilization; accordingly, the roles of psychological capital and coping styles may differ from those observed in other treatment contexts. Second, the study focuses on a specific treatment stage. The assessment time point in this study was set at mid-course radiotherapy (cumulative dose reaching 50%). At this stage, patients simultaneously experience distress from current side effects and uncertainty about subsequent treatment, with dual pressures intertwining. The “empowerment → transformation” pattern of the chain mediating pathway may be particularly relevant at this phase. Third, this study proposes a more integrated conceptual framework. To our knowledge, this study is the first to integrate psychological capital, as an internal resource, and coping styles, as behavioral strategies, into a serial mediating model. In doing so, it is consistent with the possibility that external resources may be linked to psychosomatic outcomes through two sequential stages—psychological empowerment and behavioral response—thereby extending previous work that has largely focused on single mediators or direct associations. From a theoretical perspective, this finding integrates conservation of resources theory with the transactional model of stress and coping. Specifically, social support, as an external resource, may help patients build psychological capital, activate internal strengths, and promote the selection of adaptive coping strategies, which is consistent with a theoretically proposed pathway from external resources to internal resources and then to behavioral strategies. Although the chain mediating pathways were statistically significant, their effect proportions were relatively small, accounting for 7.1% of the total effect in Model 1 and 4.03% in Model 2. Therefore, these chain mediation effects should not be interpreted as dominant explanatory mechanisms. Rather, they indicate modest but clinically meaningful and actionable pathways through which social support may be associated with psychosomatic status via psychological capital and coping styles. From a practical perspective, the clinical value of these findings lies not in the magnitude of the indirect effects alone, but in identifying specific and modifiable psychosocial targets for screening and intervention during mid-course radiotherapy. At the clinical level, this pathway suggests that psychological capital cultivation and coping-skills training may be useful components of supportive care, but they should be integrated with broader clinical strategies, including symptom management, socioeconomic support, and stage-specific care.

### 4.3. Limitations and Future Directions

This study has several limitations. First, the cross-sectional design precludes causal inferences; future longitudinal studies are needed. Because all main variables were measured at a single time point during mid-course radiotherapy, the temporal ordering among social support, psychological capital, coping styles, and psychosomatic status cannot be established. Therefore, the mediation models should be interpreted as association-based models rather than evidence of causal mechanisms. Second, single-center recruitment and the exclusion of patients with an expected survival of less than one year limit the generalizability of the findings, and multicenter studies covering different cancer types and patients with greater clinical fragility or palliative radiotherapy needs are warranted. Because convenience sampling was used and participant characteristics were not formally compared with those of the broader source population, potential selection bias cannot be ruled out. Although head and neck tumors were excluded to reduce one important source of heterogeneity related to distinct functional and psychosomatic burdens, the remaining sample still included multiple cancer types with different pathophysiological mechanisms, clinical trajectories, symptom profiles, prognoses, and treatment goals. This diagnostic heterogeneity may have introduced additional variability and should be considered when interpreting the findings. Moreover, some demographic and clinical subgroups had small sample sizes, which may reduce confidence in subgroup comparisons; therefore, these subgroup findings should be interpreted with caution. In addition, future studies may further examine whether employment status and number of children are associated with specific MSPSS dimensions, such as family, friend, and significant other support. Third, although validated scales were used, self-report data may still be subject to common method bias and social desirability. Although Harman’s single-factor test did not indicate a single dominant factor, it is only a preliminary diagnostic method, and common method bias cannot be completely ruled out. In addition, psychosomatic status was assessed using the PSSCP, a cancer-specific instrument developed and validated in Chinese populations. However, this study did not include internationally widely used comparison measures of distress, symptom burden, or quality of life; therefore, cross-study comparability with international literature may be limited. Future research should incorporate objective measures, such as physiological parameters and behavioral records, as well as internationally validated patient-reported outcome measures, to further examine these observed associations and indirect pathways.

### 4.4. Clinical Implications

The findings of this study have several practical implications for supportive strategies for improving the psychosomatic status of cancer patients during mid-course radiotherapy. First, patients’ social support should be systematically assessed. In radiotherapy departments, the mid-course point at which patients have received approximately 50% of the planned radiation dose may serve as a practical checkpoint for brief psychosocial screening. This screening could be incorporated into routine weekly on-treatment reviews and include perceived social support, psychological capital, coping risk, and psychosomatic symptoms. Patients with low perceived support, low psychological capital, high negative coping, or prominent psychosomatic symptoms could then be referred to psycho-oncology services, nurse-led counseling, peer support, or structured coping-skills interventions. At mid-course radiotherapy, the level of social support from family, healthcare providers (including physicians, radiation therapists, and nurses), and peers should be comprehensively evaluated to identify weaknesses in support networks, thereby providing a basis for subsequent interventions. Second, interventions could be implemented to strengthen psychological capital. Techniques such as mindfulness training, cognitive restructuring, and goal-setting can be used to enhance patients’ self-efficacy and hope. Narrative therapy may help patients reconstruct their understanding of illness-related adversity and strengthen psychological resilience [63,64]. Third, integrated training in psychological capital and coping skills could be promoted. For example, patients can be guided to develop an “activity-rest” plan when fatigued (problem-solving coping) and to actively seek support when experiencing emotional distress (help-seeking coping), thereby translating a positive mindset into concrete behavioral patterns and forming a virtuous cycle of “psychological empowerment → behavioral transformation.”

## 5. Conclusions

The present study found that social support was associated with better psychosomatic status among cancer patients undergoing mid-course radiotherapy, with psychological capital and coping styles showing both independent and serial mediating roles. These findings are consistent with a theoretically proposed pathway characterized by “resource empowerment → behavioral response.” Clinically, greater attention may be warranted to assessing social support in patients undergoing mid-course radiotherapy, and integrated interventions that combine psychological capital cultivation with coping skills training may be considered to support patients’ psychosomatic status.

## Figures and Tables

**Table 1:** Demographic and clinical characteristics of participants (N = 327).

Variable	Category	n (%)	*M* ± *SD*	*t/F*	*p*
Sex	Male	131	55.04 ± 15.07	−1.219	0.224
	Female	196	57.04 ± 14.21		
Age (years)	18–44	49	60.24 ± 15.16	2.997	0.051
	45–59	180	56.34 ± 13.44		
	≥60	98	54.05 ± 15.93		
Educational level	Primary school	114	58.30 ± 15.98	1.797	0.148
	Junior high school	98	56.44 ± 12.41		
	Senior high school	82	54.57 ± 12.71		
	College or above	33	52.67 ± 18.71		
Marital status	Divorced/widowed	11	64.36 ± 12.64	2.615	0.075
	Single	6	63.50 ± 5.47		
	Married	310	55.81 ± 14.65		
Employment status	unemployed	245	57.23 ± 13.99	2.011	0.046 *
	Employed	82	53.27 ± 15.91		
Number of children	No children	6	63.50 ± 5.47	2.981	0.032 *
	1 child	133	57.41 ± 14.32		
	2 children	138	56.67 ± 14.59		
	3 or more children	50	51.06 ± 14.95		
Household registration	Urban	112	57.55 ± 14.21	1.179	0.239
	Rural	215	55.55 ± 14.74		
Income per capita	<3000 RMB	127	57.69 ± 14.46	5.887	0.003 **
	3000–5000 RMB	164	56.80 ± 13.68		
	>5000 RMB	36	48.58 ± 16.85		
Insurance type	Employee insurance	121	54.88 ± 15.70	0.95	0.39
	Resident insurance (including NCMS)	196	56.93 ± 14.05		
	Self-paid	10	59.10 ± 9.27		
Diagnosis	Bone tumors	16	49.19 ± 16.24	2.078	0.055
	Thoracic tumors	72	56.64 ± 14.18		
	Abdominal tumors	85	58.96 ± 14.78		
	Pelvic tumors	16	62.31 ± 15.12		
	Breast tumors	98	54.91 ± 13.69		
	Hematologic and lymphatic tumors	7	56.14 ± 12.94		
	Others	33	52.79 ± 15.23		
TNM stage	Stage I	31	52.45 ± 16.25	2.756	0.043 *
	Stage II	80	53.99 ± 14.03		
	Stage III	120	56.30 ± 14.75		
	Stage IV	96	59.26 ± 13.81		

Note: Income per capita = Monthly household income per capita; NCMS = New Rural Cooperative Medical Scheme; TNM = Tumor–Node–Metastasis. Values are presented as n (%) or mean ± standard deviation. The PSSCP values represent mean total scores within each demographic or clinical subgroup. The *p*-values refer to group comparisons of PSSCP total scores across demographic and clinical subgroups. Higher PSSCP scores indicate poorer psychosomatic status. * *p* < 0.05; ** *p* < 0.01.

**Table 2:** Mean scores and correlation analysis among variables (N = 327).

Variable	*M* ± *SD*	1	2	3	4	5
1 SC	54.73 ± 14.81	1				
2 PPC	114.84 ± 32.38	0.392 ***	1			
3 PC	21.78 ± 5.53	0.485 ***	0.475 ***	1		
4 NC	52.65 ± 14.42	−0.374 ***	−0.346 ***	−0.295 ***	1	
5 CPS	56.24 ± 14.57	−0.428 ***	−0.416 ***	−0.456 ***	0.360 ***	1

Note: SC = Social Support; PPC = Positive Psychological Capital; PC = Positive Coping (Confrontation); NC = Negative Coping; CPS = Cancer Psychosomatic Status. Pearson correlation coefficients are presented. *** *p* < 0.001.

**Table 3:** Multiple linear regression predicting psychosomatic status.

Independent Variables	*B*	*SE*	*β*	*t*	*p*	*F*	Adjusted *R*^2^
(Constant)	5.423	0.414		13.091	<0.001 ***	21.24 ***	0.33
Employment status	−0.171	0.097	−0.082	−1.766	0.078		
Number of children	−0.109	0.056	−0.089	−1.938	0.054		
Income per capita	−0.129	0.064	−0.092	−2.000	0.046 *		
Cancer stage	0.070	0.044	0.073	1.584	0.114		
PPC	−0.112	0.040	−0.153	−2.818	0.005 **		
PC	−0.273	0.064	−0.237	−4.258	<0.001 ***		
NC	0.170	0.061	0.142	2.78	0.006 **		
SC	−0.135	0.040	−0.183	−3.337	0.001 **		

Note: Dependent Variable: PSSCP total mean score; Income per capita = Monthly household income per capita; SC = Social Support; PPC = Positive Psychological Capital; PC = Positive Coping (Confrontation); NC = Negative Coping; * *p* < 0.05, ** *p* < 0.01, *** *p* < 0.001.

**Table 4:** Bootstrap analysis for significance testing of mediating effects.

	Path	Estimate	SE	95% CI Lower	95% CI Upper	*p*	Ratio (%)
Model 1	Total effect	−0.521	0.053	−0.615	−0.409	0.000	100
	Direct effect	−0.288	0.075	−0.429	−0.134	0.000	55.30
	Ind1: Social support → Psychological capital → Psychological status	−0.112	0.037	−0.193	−0.048	0.001	21.50
	Ind2: Social support → Positive coping → Psychological status	−0.085	0.029	−0.152	−0.037	0.000	16.30
	Ind3: Social support → Psychological capital → Positive coping → Psychological status	−0.037	0.013	−0.068	−0.016	0.000	7.10
	Total indirect effect	−0.234	0.043	−0.328	−0.157	0.000	44.90
Model 2	Total effect	−0.520	0.053	−0.614	−0.407	0.000	100
	Direct effect	−0.314	0.074	−0.458	−0.163	0.000	60.40
	Ind1: Social support → Psychological capital → Psychological status	−0.128	0.037	−0.212	−0.068	0.000	24.60
	Ind2: Social support → Negative coping → Psychological status	−0.057	0.027	−0.119	−0.011	0.015	10.90
	Ind3: Social support → Psychological capital → Negative coping → Psychological status	−0.021	0.011	−0.051	−0.004	0.012	4.03
	Total indirect effect	−0.206	0.042	−0.293	−0.127	0.000	39.53

## Data Availability

The datasets generated and/or analyzed during the current study are not publicly available because they contain potentially identifiable patient information, but are available from the corresponding author upon reasonable request.

## Abbreviations

The following abbreviations are used in this manuscript:

AGFI	Adjusted Goodness-of-fit Index
ANOVA	Analysis of Variance
AVE	Average Variance Extracted
CCMQ	Cancer Coping Modes Questionnaire
CFI	Comparative Fit Index
CMB	Common Method Bias
COR	Conservation of Resources
CPS	Cancer Psychosomatic Status
CR	Composite Reliability
GFI	Goodness-of-fit Index
IFI	Incremental Fit Index
Income Per Capita	Monthly Household Income Per Capita
MSPSS	Multidimensional Scale of Perceived Social Support
NC	Negative Coping
NCMS	New Rural Cooperative Medical Scheme
PC	Positive Coping (Confrontation)
PPC	Positive Psychological Capital
PPQ	Positive Psychological Capital Questionnaire
PSSCP	Psychosomatic Status Scale for Cancer Patients
RMSEA	Root Mean Square Error of Approximation
SC	Social Support
TLI	Tucker–Lewis Index
TNM	Tumor–Node–Metastasis
